# Coinfection and Interference Phenomena Are the Results of Multiple Thermodynamic Competitive Interactions

**DOI:** 10.3390/microorganisms9102060

**Published:** 2021-09-29

**Authors:** Marko Popovic, Mirjana Minceva

**Affiliations:** Biothermodynamics, TUM School of Life Sciences, Technical University of Munich, Maximus-von-Imhof-Forum 2, 85354 Freising, Germany; mirjana.minceva@tum.de

**Keywords:** biothermodynamics, Gibbs energy, susceptibility, permittivity, infection

## Abstract

Biological, physical and chemical interaction between one (or more) microorganisms and a host organism, causing host cell damage, represents an infection. Infection of a plant, animal or microorganism with a virus can prevent infection with another virus. This phenomenon is known as viral interference. Viral interference is shown to result from two types of interactions, one taking place at the cell surface and the other intracellularly. Various viruses use different receptors to enter the same host cell, but various strains of one virus use the same receptor. The rate of virus–receptor binding can vary between different viruses attacking the same host, allowing interference or coinfection. The outcome of the virus–virus–host competition is determined by the Gibbs energies of binding and growth of the competing viruses and host. The virus with a more negative Gibbs energy of binding to the host cell receptor will enter the host first, while the virus characterized by a more negative Gibbs energy of growth will overtake the host metabolic machine and dominate. Once in the host cell, the multiplication machinery is shared by the competing viruses. Their potential to utilize it depends on the Gibbs energy of growth. Thus, the virus with a more negative Gibbs energy of growth will dominate. Therefore, the outcome can be interference or coinfection, depending on both the attachment kinetics (susceptibility) and the intracellular multiplication machinery (permittivity). The ratios of the Gibbs energies of binding and growth of the competing viruses determine the outcome of the competition. Based on this, a predictive model of virus–virus competition is proposed.

## 1. Introduction

In 2020, during the COVID-19 pandemic, a surprisingly low number of influenza cases was reported worldwide [1,2,3]. Many are wondering whether this is a result of intense vaccination against influenza, the application of epidemiological measures, such as masks and social distancing, or interference [2].

Viral interference is a phenomenon where a virus can prevent or partially inhibit infection with another virus within the same host [4,5,6], implying infection by two viruses consecutively. However, it is important to underline that simultaneous infection with two viruses can also result in interference or coinfection. Interference is an important phenomenon that influences the dynamics of spreading of viruses that co-circulate in a population. It was observed that an epidemic caused by one virus can be suppressed by a co-circulating virus [3,7]. Therefore, it would be beneficial to have a model that would allow us to better understand and predict the outcome of viral interaction.

Various living species surround and interact with each other. Infection represents a biological, physical and chemical interaction between microorganisms and a host organism, leading to host cell damage [8,9,10]. Viral infections occur as a consequence of interactions of viruses and their hosts [9,11,12,13,14,15]. Except *virus–host competitive* interaction, there can also be *virus–virus competitive* interactions [16]. When multiple pathogens circulate at the same time, this can lead to:

(a) cooperative forms (co-infections), or

(b) competitive forms (interference) of virus–virus–host interactions [16].

Mixed infections of viruses are not uncommon, and complex pathophysiological changes can result from such infections [17]. *Co-infections* can occur when two or more pathogens are in circulation and have the same seasonal pattern or overlap [16]. *Competitive forms* of virus–virus–host interactions (interference) occur when two or more viruses are in circulation but one virus interacts more efficiently with the host and, thus, prevents the other virus from infecting the same host [18], or when two viruses share the same antigen (or have very similar antigens), so the immune answer to one virus inhibits infection with another virus.

Interaction between two viruses in one host organism can be considered at two levels:

(1) Macroscopic—considering virus–virus–host interaction within a population as an epidemiological phenomenon.

(2) Microscopic—considering virus–virus–host interaction within a cell/tissue/organism as a chemical, physical and biological phenomenon.

Both approaches are necessary to analyze virus–virus–host interactions. Epidemiological data on the spreading of two or more viruses during a season can indicate that there is interference between viruses. The microscopic approach can offer an explanation of the mechanism that leads to interference. Thus, the microscopic and macroscopic approaches are complementary.

Interference has been known for a long time on a macroscopic, epidemiological level. Epidemiological data are readily available and have been collected for many years, making them more accessible than microscopic data that have to be collected in expensive experiments. Except for the high price, experiments are also hindered by difficulties in obtaining samples that are pure enough in amounts that are sufficient for analysis.

Spreading of a virus can be interrupted by an epidemic of another virus [5]. Dietz [19] discussed an analytical model for the competition of two interfering virus populations in a community. In case of large virus populations and intense disease transmission, different viruses must compete for resources. This competition was seen in a series of studies in West Bengal, India, and in Nepal, where competition for space led to the localization of adenovirus types [20]. Nickbakhsh et al. [7] reported comparative prevalence of virus interactions detected among patients from 2005 to 2013 in Glasgow, UK.

The evidence from recent surveillance data in Hong Kong suggests that viral interference during the epidemics of influenza viruses and other common respiratory viruses might affect the timing and duration of subsequent epidemics of certain or several viruses [21]. Three reports showed data suggesting that the autumn rhinovirus epidemic interrupted and delayed the spread of the 2009 H1N1 pandemic, Influenza A, in Europe [5]. However, during the influenza season, numerous strains of influenza A and B viruses can co-circulate within populations [4]. It is clear that various strains of the same virus can co-circulate in populations, while epidemics made by different viruses more often lead to interference. In the northern hemisphere, mid-December is usually the beginning of the annual cold and flu season. The epidemic is usually caused in this period by the respiratory syncytial virus and the rhinovirus, and later, in mid-February, by Influenza A and B [3]. However, in 2020, the COVID-19 pandemic surged in almost all countries, while the levels of many common seasonal infections were extremely low [3]. Thus, interference is obviously taking place. The only virus that has not been halted by COVID-19 is the rhinovirus [3]. Why has the COVID-19 pandemic halted the expected epidemics caused by respiratory syncytial virus and influenza virus, but not that caused by rhinovirus?

Viral interference represents, at the microscopic level, the inhibition of viral reproduction (and host damage) caused by previous exposure of susceptible cells to another virus [4]. Infection of an organism, whether plant, animal, or bacterium, with a virus can prevent or partially inhibit infection with another virus within the same host [4]. Ojosnegros et al. [22] considered an infection of cells with two viruses, a colonizer and a competitor. The two names come from ecology. The colonizer strain multiplies faster when present alone, making it the best at colonizing empty habitats [22,23]. On the other hand, if two or more species are present in a habitat, the species with higher fitness is the competitor and will dominate over the other species [22,23]. In cells co-infected with colonizer and competitor strains of the foot-and-mouth disease virus, viral interference resulted in reduced cell killing (damage), as slow-growing competitors replaced fast-growing colonizers [22]. Bovine viral diarrhea viruses (BVDV) are classified into BVDV-1 and BVDV-2 species. Competitive exclusion (interference) was observed as the BVDV-2a strains dominated and excluded the BVDV-1a and BVDV-1b strains [24]. Pepin et al. [25] studied the interference of two dengue virus strains, following viral population dynamics in cultured mosquito cells. The competition led to a decrease in viral populations, which was asymmetric between the two strains [25]. These phenomena represent interference appearing as a consequence of competition between viruses for cell metabolic machinery.

Viruses compete not only for host metabolic machinery, but also to enter the cell first. Spike proteins of both SARS-CoV-2 and SARS-CoV bind to ACE2 before entering the cell for replication. Wrapp et al. [26] suggested that the S-protein of SARS-CoV-2 binds to the ACE2 receptor more strongly than SARS-CoV, associating this with greater severity of the former. Casasnovas and Springer [27] determined the thermodynamic properties of the binding of the rhinovirus to its host cell, using kinetic methods and surface plasmon resonance. The Gibbs energy and enthalpy of the binding were interpreted in terms of the influence of virus stability and temperature on binding affinity [27].

Even though much information has been gathered on interference, many things remain unknown. It is unknown whether the outcome of interference is determined by virus attachment and entry, or intracellular multiplication [15,28,29,30]. Moreover, it would be of great interest to learn whether the nucleic acid of a virus can also interfere with the multiplication of another virus [28,31]. A theory of the mechanism of viral interference must be based on chemical evidence [28].

**Figure 1:**
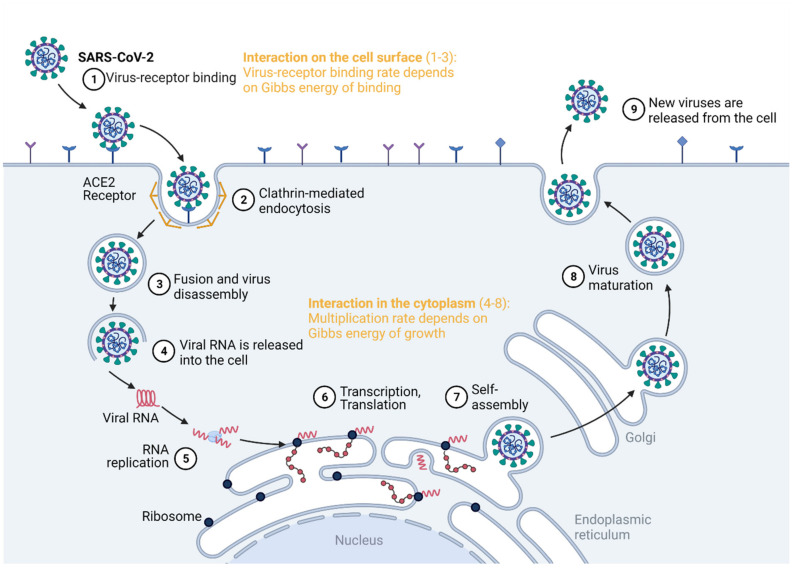
Virus–virus interactions can be quantified by the Gibbs energies of binding and growth. The Gibbs energy of binding quantifies the competition for cell entry, while the Gibbs energy of growth quantifies the ability to hijack the cell metabolism. Created with BioRender.com.

Virus competition occurs at the cell surface and in the cytoplasm, as summarized in Figure 1. The virus enters the host cell by binding to a specific receptor [15]. This is a virus–host interaction that takes place at the cell surface, which can be quantified by the Gibbs energy of binding [27,32,33,34]. Once inside the cell, the virus interacts with it in the cytoplasm, hijacking the biosynthetic pathways of the host cell [9,11,35]. This represents the intracellular virus–host interaction [36,37], quantified by the Gibbs energy of growth [9,10,38,39]. Thus, there is only one biosynthetic machine, but there are two or more potential users: the host and the virus(es). If two viruses interact with a single host, the virus with a more negative Gibbs energy will perform binding, entrance and multiplication at a greater rate than the other virus. Thus, it will be able to overtake the metabolic machinery and resources from the host and the other virus.

In mixed infection, two different viruses use different receptors to enter the host cell, but they compete for one metabolic machinery for their multiplication, as well as for resources. The two viruses must also compete with the host cell for its own metabolic pathways, but its Gibbs energy of growth is much less negative than that of the viruses [9,10], implying more spontaneous virus multiplication. The rate of viral multiplication is greater than that of any host cell; therefore, if the host is susceptible, any virus wins [9,10]. The virus with a more negative Gibbs energy wins the race and takes it all (metabolic machinery and substrates) [9].

The aim of this paper is to explore interference at the microscopic level and try to shed light on the molecular mechanism of interference. The biothermodynamic approach will be used to analyze the pathogen–pathogen–host interactions (interference and coinfection) between various virus species that potentially cause infections and epidemics in humans. Viral interference may be not one phenomenon, but many [28]. Thus, interference can be a consequence of multiple interactions taking place on the cell surface and in its cytoplasm. The hypothesis of this research is that both the Gibbs energy of binding and the Gibbs energy of growth determine susceptibility and permittivity, and hence, the outcome of interference.

## 2. Methods

### 2.1. Viruses Considered in This Study

This study analyzes the virus–virus interactions of human respiratory viruses. The considered viruses include adenovirus, influenza, parainfluenza, rhinovirus and SARS-CoV-2. The analysis was conducted, taking into account virus pairs, by comparing the thermodynamic properties of binding and growth.

### 2.2. Gibbs Energy of Binding from Dissociation Constants

Gibbs energies of virus–receptor binding were calculated from binding constants, which, in turn, were calculated from dissociation constants reported in the literature. The strength of virus attachment to host cell receptors is often expressed in terms of dissociation constants [40], modeling the process of binding as a protein–ligand interaction [41]. Dissociation constants for various viruses and their receptors were taken from the literature [42,43,44,45] and are summarized in Table 1.

The dissociation constants were used to find binding constants, *K_b_*, using the equation

(1) $$K_{b}=\frac{1}{K_{d}}=\frac{k_{on}}{k_{off}}$$

where *K_d_* is the dissociation constant, while *k_on_* and *k_off_* are kinetic rate constants that account for the forward binding and reverse unbinding (or dissociation) reaction, respectively [41]. Finally, binding constants were used to calculate the Gibbs energy of binding, Δ*_b_G*^0^**, through the equation

(2) $$\Delta_{b}G^{0}=-RT\ln K_{b}$$

where *R* is the universal gas constant and *T* is the temperature. The Δ*_b_G*^0^** values were calculated at the physiological temperature of 37 °C (310.15 K).

### 2.3. Elemental Composition of Viruses

The elemental compositions of all viruses, except SARS-CoV-2, were calculated based on molecular composition, as described in [9]. Molecular compositions of viruses were taken from the literature [50,51]. Molecular composition was first converted from mass fraction into mole fraction form through the equation

(3) $$x_{i}=\frac{w_{i}/M_{i}}{\sum_{j}w_{j}/M_{j}}$$

where *M_i_* is the molar mass in the empirical formula of molecular component *i*, and *x_i_* and *w_i_* are the mole fraction and mass fraction of molecular component *i*, respectively. The summation is over all molecular components. The mole fractions of molecular components were then used to find the empirical formula of viruses using the equation

(4) $$n_{J}=\sum_{i}x_{i}n_{i,J}$$

where *n_J_* is the number of moles of element *J* in the empirical formula of the biomass, and *n_i,J_* is the number of moles of element *J* in the empirical formula of molecular component *i*. The information about molecular components of the virus, including *n_i,J_* and *M_i_*, is given in the Supplementary Material (Table S3).

The elemental composition of SARS-CoV-2 was calculated through the atom counting method. The atom counting method gives the elemental composition of any virus based on its nucleic acid sequence, protein sequences and morphology, as described in [9,10]. The nucleic acid sequence was taken from the NCBI database (Sequence ID: NC_045512.2). Since each nucleotide residue has a well-defined elemental composition, the number of atoms of each element in the nucleic acid was found by going along the sequence and adding atoms of each element coming from each residue. The atom counting was conducted using custom-made software. Similarly, the NCBI database was used to obtain structural protein sequences, including the nucleoprotein (Sequence ID: QIK50455.1), membrane protein (Sequence ID: QHR63293.1) and spike protein (Sequence ID: QHR63290.2). Since each amino acid residue has a known elemental composition, the composition of the proteins was obtained by following the sequence and adding atoms of each element. However, unlike with the nucleic acid, each structural protein was present in multiple copies in the virions. Thus, the number of atoms in each protein was multiplied by the number of copies of the protein (2368 nucleoprotein, 1184 membrane protein and 222 spike protein copies per virion in total [10,52,53]). Finally, the atoms coming from nucleic acid and proteins were summed. The total number of atoms of each element was divided by the number of carbon atoms to obtain an empirical formula, which has the form CH_nH_O_nO_N_nN_P_nP_S_nS_. The elemental compositions of the analyzed viruses are given in Table 2.

### 2.4. Standard Thermodynamic Properties of Viruses

The elemental compositions of the viruses were used to determine their thermodynamic properties, as described in [9,10,54]. The equations used are given in Table 3.

The enthalpy of the viruses was determined using the Patel–Erickson equation and Hess’ law. First, the elemental compositions of the viruses were used to find the number of electrons transferred to oxygen during complete combustion, *E*, using Equation (5) [55,56]. The number of electrons transferred to oxygen was then used to find standard enthalpy of combustion of virus live matter, Δ*_C_H*^0^**, through the Patel–Erickson equation (Equation (6)) [55,56]. The standard enthalpy of combustion was then converted into the standard enthalpy of formation of live matter, Δ*_f_H*^0^**(*Bio*), using Hess’ law (Equation (7)) and the standard thermodynamic properties of oxides [57,58,59]. Similarly, the standard molar entropy of virus live matter, *S_m_*^0^**(*Bio*), was calculated using the Battley equation (Equation (8)) [60]. The Battley equation can also be modified to yield the standard entropy of formation of virus live matter, Δ*_f_S*^0^**(*Bio*), (Equation (9)) [60]. Finally, Δ*_f_H*^0^**(*Bio*) and Δ*_f_S*^0^**(*Bio*) were combined to find the standard Gibbs energy of formation of virus live matter, Δ*_f_G*^0^**(*Bio*), through Equation (10). Thermodynamic properties of virus live matter are given in the Supplementary Material (Table S2).

(5) $$E=4\;n_{C}+n_{H}-2\;n_{O}-0\;n_{N}+5\;n_{P}+6\;n_{S}$$

(6) $$\Delta_{C}H^{0}=-111.14\frac{kJ}{mol}\cdot E$$

(7) $$\Delta_{f}H^{0}\left(Bio\right)=n_{C}\Delta_{f}H^{0}\left(CO_{2}\right)+\frac{1}{2}n_{H}\Delta_{f}H^{0}\left(H_{2}O\right)+\frac{1}{4}n_{P}\Delta_{f}H^{0}\left(P_{4}O_{10}\right)+n_{S}\Delta_{f}H^{0}\left(SO_{3}\right)-\Delta_{C}H^{0}$$

(8) $$S_{m}^{o}\left(Bio\right)=0.187\underset{J}{\sum }\frac{S_{m}^{o}\left(J\right)}{a_{J}}n_{J}$$

(9) $$\Delta_{f}S^{0}\left(Bio\right)=-0.813\underset{J}{\sum }\frac{S_{m}^{o}\left(J\right)}{a_{J}}n_{J}$$

(10) $$\Delta_{f}G^{0}\left(Bio\right)=\Delta_{f}H^{0}\left(Bio\right)-T\;\Delta_{f}S^{0}\left(Bio\right)$$

CH_1_._7978_O_0_._4831_N_0_._2247_S_0_._0225_ + O_2_ + HPO_4_^2-^ + HCO_3_^-^ → (Bio) + SO_4_^2-^ + H_2_O + HCO_3_^-^ + H_2_CO_3_ (11)

(12) $$\Delta_{r}G^{0}=\underset{products}{\sum }\nu \;\Delta_{f}G^{0}-\underset{reactants}{\sum }\nu \;\Delta_{f}G^{0}$$

(13) $$\delta \left(\Delta_{f}H^{0}\left(bio\right)\right)=\delta \left(\Delta_{C}H^{0}\right)=0.0536\;\cdot \left|-111.14\frac{kJ}{mol}\left(4\;n_{C}+n_{H}-2\;n_{O}-0\;n_{N}+5\;n_{P}+6\;n_{S}\right)\right|$$

(14) $$\Delta_{f}S^{0}\left(Bio\right)=-0.813\underset{J}{\sum }\frac{S_{m}^{o}\left(J\right)}{a_{J}}n_{J}$$

### 2.5. Gibbs Energy of Growth

The thermodynamic properties of the growth of viruses were determined as described in [9,10,54]. The growth of viruses is manifested as population growth, i.e., multiplication. Based on the elemental compositions of the viruses, growth reactions were formulated that describe the formation of virions from nutrients (Equation (11) in Table 3). The nutrient compositions were taken from [9,10] and resemble those in human blood plasma, from which the host cells and ultimately viruses obtain their building blocks. The nutrients included amino acids (CH_1_._7978_O_0_._4831_N_0_._2247_S_0_._0225_) as the carbon, nitrogen and sulfur source, O_2_ as the electron acceptor, HPO_4_^2-^ as the phosphorus source, and HCO_3_^-^/H_2_CO_3_ buffer the as the pH regulator. More information about the nutrients can be found in the Supplementary Material (Table S4). The stoichiometric coefficients for the growth reaction (Equation (11)) are given in the Supplementary Material (Table S1). Based on the stoichiometric coefficients and the Gibbs energies of formation of viruses from Section 2.3, the Gibbs energies of growth were calculated using Equation (12). The thermodynamic properties of growth of viruses are given in the Supplementary Material (Table S2).

### 2.6. Uncertainties

The determined thermodynamic properties had an uncertainty that originated from the model used for their estimation. The Pate–Erickson equation gave the standard enthalpy of combustion of with an accuracy of 5.36% [54]. The same 5.36% uncertainty was then passed on to standard enthalpy of formation of live matter (Equation (13)) [54]. Similarly, the standard molar entropy of live matter was calculated through the Battley equation with an 19.7% uncertainty, which was then passed on to standard entropy of formation of live matter (Equation (14)) [60].

## 3. Results and Discussion

**Figure 2:**
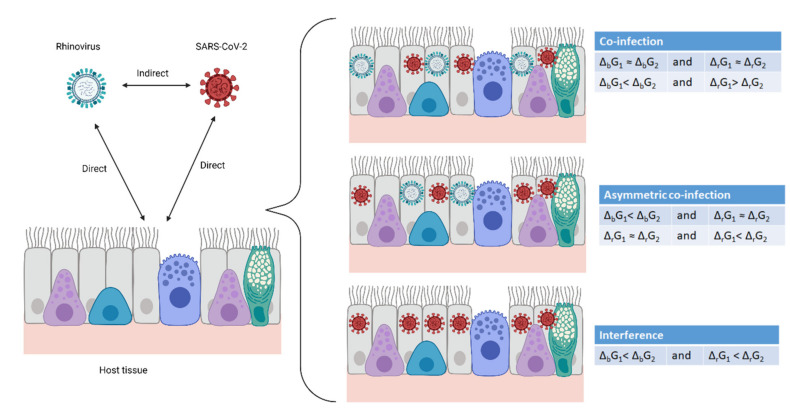
Virus–host and virus–virus interactions. Two viruses and a multicellular organism make two direct interactions—each virus with the host. In this way, the viruses interact with each other, indirectly through the host. The result can be co-infection, asymmetric co-infection and interference. Created with BioRender.com.

The interaction between a virus (system A) and its host cell (system C) is a simple two-system interaction. However, if a single cell (system C) is in contact with virus A (system A) and virus B (system B), the interaction becomes complex, consisting of two simultaneous simple interactions: A–C and B–C. Thus, virus A and virus B interact through a mediator—the host cell—with which they interact simultaneously, making the interaction transitive. The interaction can lead to three outcomes: co-infection, asymmetrical co-infection and interference, as shown in Figure 2. Co-infection represents independent circulation of viruses in a population. In asymmetric co-infection, two viruses circulate, but one dominates. Interference implies that one virus completely suppresses another. These interactions are fundamentally chemical, and hence, obey the laws of chemical thermodynamics and kinetics.

### 3.1. Gibbs Energy Determines the Outcome of Virus–Virus–Host Interactions

Two viruses infecting the same host interact at two sites, the cell membrane and the cytoplasm, as summarized in Figure 1. At both sites, the interaction is quantified by a Gibbs energy. First, in order to infect a cell, a virus must enter through the cell membrane. The attachment of the virus to the membrane receptor, or the susceptibility [61], is quantified by the Gibbs energy of binding, influencing the binding rate, as will be discussed below [27,32,33,34,40,46,62]. Once the virus enters the cytoplasm, it performs disassembly, a process that is the opposite of self-assembly. The nucleocapsid disassembles into nucleic acid and protein components. Next, the virus performs replication and transcription. Then, it must hijack the metabolism of the host cell in order to perform translation. This process is also known as permittivity [61] and is characterized by the Gibbs energy of growth [9,10]. At both the entrance and multiplication steps, the two viruses compete through the dissipation of Gibbs energy. The more the Gibbs energy is dissipated, the more negative the Gibbs energy change for the process and the greater its rate [63].

The rates of both entrance and multiplication of viruses are proportional to their driving forces—Gibbs energy dissipation. The rate of a process depends on both thermodynamic and kinetic factors, which are summarized by the linear phenomenological equation

(15) $$r_{p}=-\frac{L_{p}}{T}\Delta_{p}G$$

where *T* is the temperature, *r_p_* is the rate of process p, *L_p_* is a phenomenological coefficient for process p, and Δ*_p_G* is the Gibbs energy change for process p [63,64]. The linear phenomenological equation has been applied to a wide range of chemical, physical and biological processes [38,63,64,65,66]. Moreover, it has been applied to virus multiplication in the cytoplasm [9,10]. The virus multiplication rate in the cytoplasm, *r_r_*, is proportional to Gibbs energy of growth, Δ*_r_G*, of the virus [9,10]. Thus, the Gibbs energy of growth can be used to quantify the interaction of viruses in the host cell cytoplasm. Similarly, the rate of virus attachment to the host cell, *r_b_*, can be quantified by the Gibbs energy of binding, Δ*_b_G* [27,32,33,34,40,46,62], according to the linear phenomenological equation [63,64]. At the molecular level, a more negative Gibbs energy of binding implies stronger attachment of the virus to the receptor, which in turn implies more efficient entrance into the cell [26,40,46]. This gives the virus a head start in terms of multiplying and, therefore, facilitates multiplication. Thus, the Gibbs energies of binding and growth determine both virus attachment (susceptibility) and multiplication in the cytoplasm (permittivity), respectively.

Viruses are obligate intracellular parasites [67]. Before multiplication, the virus must enter the host by binding to the specific receptor [15]. The Gibbs energy of binding characterizes the susceptibility of the host cells to various viruses [27,32,33,34,40,62]. The Gibbs energies of binding of the 14 viruses considered in this work are summarized in Table 1. They were obtained from three sources: (A) from dissociation constants reported in the literature, using Equations (1) and (2); (B) from enthalpies, Δ*_b_H*, and entropies, Δ*_b_S*, of binding reported in the literature, using the equation Δ*_b_G* = Δ*_b_H* − *T* Δ*_b_S*; (C) directly from the literature. Gibbs energies of binding are of the same order of magnitude, but slightly differ between various virus species. If two viruses simultaneously infect the same host, the virus with a more negative Gibbs energy of binding attaches and enters the host cell more efficiently. This head start allows one virus to hijack the host cell metabolic machinery more quickly, giving it an advantage in terms of multiplication.

Except for receptor binding, interference also depends on the ability of the two viruses to multiply inside the cell—permittivity. Permittivity is characterized by the Gibbs energy of growth [9,10]. The Gibbs energies of growth of various viruses were calculated for the first time in this paper, using the equations from Table 3, and are given in Table 2.

Gibbs energies of growth determine multiplication rates of various viruses, according to the linear equation, because viral multiplication consists of several chemical processes. Thus, they influence virus–virus–host interactions (interference). A virus with a lower Gibbs energy of growth (multiplication) is able to suppress the multiplication of another virus. Table 4 presents the Gibbs energies of binding and growth of several respiratory viruses that can perform interference or co-infections.

### 3.2. The Arena: Theoretical Predictions and Experimental Evidence

Similarly to gladiators in the arena, viruses interact competitively inside cells, fighting for survival. The virus with lower Gibbs energies of binding and growth dominates. Whether the interaction of two viruses will result in co-infection or interference depends on the Gibbs energies of binding and growth, which characterize both viruses. The discussion below is about the outcomes of interactions of various virus pairs on a single host. The thermodynamic properties and outcomes on which the analysis is based are summarized in Table 4. Two assumptions are made. First, interference due to immune response is not considered in this paper. Second, the inoculation doses of both viruses are the same. The immune response as a reason for interference lays in the domain of immunology and not thermodynamics. This paper considers the potential biothermodynamic mechanism as the cause of interference. However, it would be interesting to expand the discussion to the thermodynamic mechanisms of the immune response. Interference as a phenomenon is possible in organisms that lack an immune system. An example is interference in bacteriophages.

**Rhinovirus vs. SARS-CoV-2:** The rhinovirus interacts with SARS-CoV-2 at the cell surface receptor and at the intracellular level. In simultaneous infection by both viruses, the rhinovirus enters the host cell at a lower rate, since the Gibbs energy of binding of the rhinovirus is −28 to −39 kJ/mol, while that of SARS-CoV-2 is −42.2 to −51.4 kJ/mol. However, the rhinovirus multiplies at a higher rate, since the Gibbs energy of growth of the rhinovirus is −197 kJ/mol, while that of SARS-CoV-2 is −165 kJ/mol. Since the SARS-CoV-2 has a more negative Gibbs energy of binding, it will enter host cells faster than the rhinovirus. Even though SARS-CoV-2 enters host cells faster due to its more negative Gibbs energy of binding, the rhinovirus will be able to hijack cell metabolism more effectively, due to its more negative Gibbs energy of growth. This implies that during infection, both viruses will be present in multicellular organisms. This conclusion is supported by the observation that, in 2020, rhinovirus was able to constitute an epidemic in parallel with the SARS-CoV-2 pandemic [3].

**Influenza vs. SARS-CoV-2:** SARS-CoV-2 dominates over influenza, due to its having more negative Gibbs energies of binding and growth. In simultaneous infection by both viruses, the influenza virus enters the host cell at a lower rate, since the Gibbs energy of binding of the influenza virus is −32.6 to −33.7 kJ/mol, while that of SARS-CoV-2 is −42.2 to −51.4 kJ/mol. The influenza virus multiplies at a lower rate because its Gibbs energy of growth is −83 to −87 kJ/mol, while that of SARS-CoV-2 is −165 kJ/mol. As a result, the influenza virus enters host cells more slowly and multiplies more slowly. Therefore, SARS-CoV-2 dominates in the population and the outcome is interference. Indeed, the incidence of influenza reported to the WHO was low in 2020 [1]. The incidence of seasonal influenza was unusually low at the southern hemisphere [2]. Moreover, southeast Asia, South America and Africa reported zero cases of seasonal flu [2].

Influenza and SARS-CoV-2 use different receptors for cell entry: sialic acid and ACE2, respectively. Sialic acid is more prevalent than ACE2, but the binding affinity of the S-protein of SARS-CoV-2 to ACE2 (−42.2 to −51.4 kJ/mol) is stronger than that of influenza for sialic acid (−32.6 to −33.7 kJ/mol). Thus, even though sialic acid is more prevalent than ACE2, SARS-CoV-2 has an advantage over influenza due to its more negative Gibbs energy of binding. Moreover, since SARS-CoV-2 and the influenza virus both infect cells of the respiratory system, they compete for resources [68]. Having lower Gibbs energies of binding and growth, SARS-CoV-2 has a competitive advantage over influenza. This is in agreement with the findings of Hagen [2].

**Adenovirus vs. SARS-CoV-2:** In infection by both viruses, the adenovirus enters the host cell at approximately the same rate as SARS-CoV-2 since their Gibbs energies of binding are very similar (adenovirus: −45.1 to −45.7 kJ/mol; SARS-CoV-2: −42.2 to −51.4 kJ/mol). Adenovirus performs multiplication at a lower rate, since its Gibbs energy of growth is −144 kJ/mol, while that of SARS-CoV-2 is −165 kJ/mol. In case of a simultaneous infection, both viruses should circulate, but SARS-CoV-2 should dominate. This conclusion is also supported by observation. In New South Wales, the detection of adenoviruses held relatively steady throughout the southern winter, rather than crashing, as was the case for flu, or surging, as was observed for the rhinovirus [3].

**Parainfluenza vs. SARS-CoV-2:** In this case, the influenza virus enters the host cell at a lower rate, since the Gibbs energy of binding of the parainfluenza virus is −24.0 to −27.8 kJ/mol, while that of SARS-CoV-2 is −42.2 to −51.4 kJ/mol. The parainfluenza virus multiplies at a lower rate, because its Gibbs energy of growth is −86 kJ/mol, while that of SARS-CoV-2 is −165 kJ/mol. In that case, the parainfluenza virus enters host cells more slowly and multiplies more slowly. Therefore, SARS-CoV-2 dominates in the population and the outcome is interference. Cases of COVID-19 and parainfluenza-4 virus co-infection are very rare [69].

**Influenza vs. adenovirus:** The influenza virus enters the host cell at a lower rate because the Gibbs energy of binding of the influenza virus is −32.6 to −33.7 kJ/mol, while that of the adenovirus is −45.1 to −45.7 kJ/mol. The influenza virus performs multiplication at a lower rate, since the Gibbs energy of growth of the influenza virus is −83 to −87 kJ/mol, while that of the adenovirus is −165 kJ/mol. Thus, the adenovirus should dominate in case both viruses are present and the outcome is interference. This conclusion is supported by the results of Nickbakhsh et al. [7], who compared the prevalence of the adenovirus and influenza B viruses from 2005 to 2013. The adenovirus began an epidemic during the influenza B epidemic [7]. The result was an increase in the prevalence of the adenovirus and decrease in that of the influenza B virus [7]. The observed phenomenon can be explained through the more negative Gibbs energy of binding and Gibbs energy of growth of the adenovirus, which allowed it to inhibit the influenza virus.

**Influenza vs. parainfluenza:** This pair is very interesting, since both viruses have similar thermodynamic properties. In case of infection with both viruses, the parainfluenza virus enters the host cell at a lower rate because the Gibbs energy of binding of the influenza virus is −32.6 to −33.7 kJ/mol, while that of parainfluenza is −24.0 to −27.8 kJ/mol. Both viruses multiply at the same rate, since the Gibbs energy of growth of the influenza virus is −83 to −87 kJ/mol, while that of parainfluenza is −86 kJ/mol. Thus, since the rate of entrance of the influenza virus is slightly greater, a small domination of influenza is expected if both viruses are present, and the expected outcome is co-infection. This result is supported by the observation of Goto et al., [70], who claimed that the growth of the influenza A virus was followed by coinfection with the parainfluenza 2 virus. Moreover, Pinky and Dobrovolny [68] reported that the influenza virus dominates over the parainfluenza virus.

**Parainfluenza 1 vs. parainfluenza 2:** The parainfluenza 1 and parainfluenza 2 viruses perform co-infection, due to their similar Gibbs energies of binding and growth. In case of simultaneous infection by both viruses, both of the parainfluenza viruses enter host cells at approximately the same rate, because the Gibbs energy of binding of the parainfluenza 1 virus is −24.0 to −27.8 kJ/mol and that of parainfluenza 2 is −21.7 to −30.4 kJ/mol. Both viruses multiply at the same rate, because the Gibbs energy of growth of both parainfluenza viruses is −86 kJ/mol. Thus, the expected outcome is co-infection. This result is in agreement with that of Nickbakhsh et al. [7], who reported similar prevalence of parainfluenza 1 and parainfluenza 2 in mixed infections.

**Rhinovirus vs. influenza:** The rhinovirus dominates over the influenza virus due to its more negative Gibbs energies of binding and growth. The influenza virus interacts with the rhinovirus. In simultaneous infection with both viruses, the influenza virus enters host cells at a lower rate, because the Gibbs energy of binding of the influenza virus is −32.6 to −33.7 kJ/mol and that of the rhinovirus is −28 to −39 kJ/mol [27]. The influenza virus performs multiplication at a lower rate, because the Gibbs energy of growth of the influenza virus is −83 to −87 kJ/mol and that of the rhinovirus is −197 kJ/mol. Thus, the rhinovirus should dominate and the expected outcome is interference. This outcome was observed by Wu et al. [5] and Pinky and Dobrovolny [68]. In Europe, the autumn rhinovirus epidemic interrupted and delayed the spread of the 2009 H1N1 pandemic of influenza A [5]. Pinky and Dobrovolny [68] found that the rhinovirus reduces replication of the influenza virus.

**Rhinovirus vs. parainfluenza:** The rhinovirus has a Gibbs energy of binding of −28 to −39 kJ/mol [27], while that of the parainfluenza virus is −24.0 to −27.8 kJ/mol. Thus, the rhinovirus has an advantage during cell entry. Moreover, the Gibbs energy of growth of the rhinovirus is −197 kJ/mol, while that of parainfluenza is −86 kJ/mol. Thus, again, the rhinovirus multiplies faster and will dominate over the parainfluenza virus. The competition will result in interference. This result is supported by the finding that the rhinovirus reduces replication of the parainfluenza virus [68].

Interference in respiratory virus infections was analyzed by Pinky and Dobrovolny [68]. Five viruses were considered: influenza, respiratory syncytial virus, rhinovirus, parainfluenza virus and human metapneumovirus [68]. The results showed that rhinovirus, the fastest-growing virus, reduces replication of the remaining viruses during a coinfection, while parainfluenza virus, the slowest-growing virus, is suppressed in the presence of other viruses [68]. These facts can be explained by the mechanism suggested in this paper. Namely, the rhinovirus has a Gibbs energy of growth of −197 kJ/C-mol and, thus, according to Equation (3), multiplies at a high rate. On the other hand, the parainfluenza virus has a Gibbs energy of growth of −86 kJ/C-mol and, thus, multiplies at the lowest rate. Moreover, the rhinovirus enters the host faster, because it is characterized by a more negative Gibbs energy of binding, according to Equation (3). Thus, the rhinovirus enters into the cell faster and multiplies faster, preventing the parainfluenza virus from overtaking the metabolism.

Nowak et al. [71] found that amongst the patients who tested positive for SARS-CoV-2 and were also tested for other respiratory viruses (8990 patients in total), co-infection was found in only 36 (<3%). The most common co-infecting viruses with SARS-CoV-2 were found to be other viruses from the family *Coronaviridae* [71]. Obviously, because SARS-CoV-2 and other *Coronaviridae* are characterized by very similar Gibbs energies of binding and growth, they are able to co-infect.

The analysis above implies that if two viruses are present in circulation, the outcome depends on their Gibbs energies of binding and growth, as is summarized in Table 5.

If two viruses have similar Gibbs energies of binding and growth, the outcome is co-infection, since none of the viruses has an advantage. An example is parainfluenza 1 vs. parainfluenza 2. Similarly, if one virus has a more negative Gibbs energy of binding, while the other has a more negative Gibbs energy of growth, the outcome is again co-infection, since again no virus has an advantage. An example is rhinovirus vs. SARS-CoV-2. On the other hand, if both viruses have the same Gibbs energy of growth, but one virus has a more negative Gibbs energy of binding, both viruses will be present, but the one with the advantage will dominate. A situation where two viruses are present but one dominates is asymmetrical co-infection. Similarly, if both viruses have the same Gibbs energy of binding, but one has a more negative Gibbs energy of growth, the result will again be an asymmetric co-infection. Finally, if one virus has a more negative Gibbs energy of binding and a more negative Gibbs energy of growth, it will prevent the multiplication of the other virus and the result will be interference.

## 4. Conclusions

Virus–host and virus–virus–host interactions at the population level represent an epidemiological phenomenon. At the organism level, they represent a biological phenomenon. At the microorganism level, they represent a microbiological phenomenon. However, at the molecular level, pathogen–host and pathogen–pathogen–host interactions represent a chemical phenomenon that can be explained with a biothermodynamic mechanism. The interaction of viruses on the cell surface with their appropriate receptors can be considered as a chemical process, the driving force of which is the Gibbs energy of binding. Virus multiplication, which includes transcription, translation and self-assembly, also represents a chemical process, with the Gibbs energy of growth as its driving force. Thus, biothermodynamics can describe the mechanism of appearance of infection and interference of viruses. A virus with more negative Gibbs energies can more efficiently enter host cells and multiply. The paper gives an explanation of the observation that some viruses enter cells at greater rate or multiply at greater rate. A table was given summarizing the possible outcomes of pathogen–pathogen–host interactions.

Virus–host interactions are direct, while virus–virus–host interactions are indirect (transitive), occurring through the host as a mediator. The first is determined by the Gibbs energies of growth of the virus and host, while the second is determined by the Gibbs energies of binding and growth of the two viruses. The Gibbs energies of binding were calculated starting from dissociation constants, or from enthalpies and entropies of binding. The Gibbs energies of growth were calculated starting from virus molecular composition, or from capsid morphology, protein sequences and nucleic acid sequences. The Gibbs energies of binding and growth are the driving forces of virus entry into, and multiplication inside, the host cell, respectively, as can be seen from the linear phenomenological Equation (15). Thus, a virus with a more negative Gibbs energy of binding has an advantage over competing viruses when entering the host cell. However, once inside the cell, a virus with a more negative Gibbs energy of growth has an advantage over competing viruses when hijacking the host cell metabolism. Therefore, the outcome of the virus–virus competition depends on their Gibbs energies of binding and growth.

The predicted virus–virus–host interaction results were validated by means of comparison with known outcomes from the literature. The predictions and literature outcomes were in good agreement. Therefore, since the model requires no direct interaction data, it is predictive.

## Figures and Tables

**Table 1:** Gibbs energies of binding of viruses. Part (A): Gibbs energies of binding calculated from dissociation constants. The “Reference” column gives sources from which the dissociation constants were taken. Part (B): Gibbs energies of binding calculated from enthalpies and entropies of binding. The “Reference” column gives sources from which the Δ_b_H and Δ_b_S values were taken. Part (C): Gibbs energies of binding reported in the literature and references from which they were taken. Symbols: K_d_—dissociation constant; K_b_—binding affinity; Δ_b_G—Gibbs energy of binding; Δ_b_G—enthalpy of binding; Δ_b_S—entropy of binding.

(A) Binding constant				
**Name**	**K_d_ (nM)**	**K_b_ (×10^9^ M^−1^)**	**Δ_b_G (kJ/mol)**	**Reference**
Adenovirus	20 to 25	0.040 to 0.050	−45.1 to −45.7	[44]
HIV-1	0.83 to 11.6	0.0862 to 1.2	−53.9 to −47.1	[42]
HIV-2	48.5	0.0206	−43.4	[42]
Human cytomegalovirus	1.1	0.91	−53.2	[42]
Influenza (3SLN)	3200	3.1×10^−4^	−32.6	[42]
Influenza (6SLN)	2100	4.8×10^−4^	−33.7	[42]
Parainfluenza virus 1	(20.6 to 90.5) × 10^3^	(1.10 to 4.85) × 10^−5^	−24.0 to −27.8	[45]
Parainfluenza virus 2	(7.7 to 218.2) × 10^3^	(4.58 to 130) × 10^−6^	−21.7 to −30.4	[45]
Parainfluenza virus 3	(33.7 to 124.7) × 10^3^	(8.02 to 29.7) × 10^−6^	−23.2 to −26.6	[45]
Poliovirus	0.21	4.8	−57.5	[42]
Reovirus	0.5	2	−55.2	[42]
Vesicular stomatitis virus	0.15	6.7	−58.3	[42]
(B) Enthalpy and entropy				
**Name**	**Δ_b_H (kJ/mol)**	**Δ_b_S (J/mol K)**	**Δ_b_G (kJ/mol)**	**Reference**
HIV at 37°C	−263.59	−691.04	−49.26	[40]
HIV at 25°C	−198.2	−472.9	−57.2	[40]
HIV at 4 °C	−97.9	−126.01	−63.0	[40]
Arboviruses	−56.545	−125	−18	[46]
(C) Reported in the literature				
**Name**	**Δ_b_G (kJ/mol)**		**Reference**	
SARS-CoV-2	−42.2 to −51.4		[10,26,47,48,49]	
Rhinovirus	−28 to −39		[27]	

**Table 2:** Elemental compositions and Gibbs energies of growth of viruses. The elemental composition refers to empirical formulas of the form CH_nH_O_nO_N_nN_P_nP_S_nS_. The elemental compositions of the viruses were calculated as described in Section 2.2, starting from data in the “Composition reference” column. The viruses marked with an asterisk are enveloped viruses. For enveloped viruses, lipids were not included in the calculations, since they are taken from the host cell during budding and not synthesized at the ribosomes [10].

Name	n_H_	n_O_	n_N_	n_P_	n_S_	Composition Reference		Δ_r_G^0^ (kJ/C-mol)
Adenovirus	1.5386	0.3354	0.2814	0.00997	0.00551	[50]		−144 ± 43
Adenovirus	1.5386	0.3354	0.2814	0.00997	0.00551	[51]		−144 ± 43
Coliphages T2, T4, T6	1.4391	0.4708	0.3105	0.04868	0.00292	[51]		−230 ± 43
Enterovirus	1.4950	0.4080	0.2985	0.02308	0.00477	[50]		−197 ± 43
Equine encephalomyelitis	1.5493	0.3352	0.2782	0.00619	0.00575	[51]		−137 ± 43
Fowl plague	1.5852	0.3521	0.2592	0.00189	0.00568	[51]		−93 ± 43
Herpes simplex	1.5506	0.3462	0.2748	0.00887	0.00546	[51]		−129 ± 43
Influenza	1.5934	0.3540	0.2550	0.00071	0.00569	[50]		−83 ± 43
Influenza	1.5903	0.3490	0.2570	0.00071	0.00574	[51]		−87 ± 43
Orthomyxoviruses	1.5912	0.3521	0.2563	0.00088	0.00571	[50]		−86 ± 43
Paramyxoviruses	1.5912	0.3521	0.2563	0.00088	0.00571	[50]		−86 ± 43
Picornaviruses	1.4950	0.4080	0.2985	0.02308	0.00477	[50]		−197 ± 43
Poliovirus	1.4950	0.4080	0.2985	0.02308	0.00477	[50]		−197 ± 43
Poxviruses	1.5618	0.3150	0.2734	0.00241	0.00596	[50]		−122 ± 43
Reoviruses, Rotaviruses, and Caliciviruses	1.5341	0.3555	0.2839	0.01091	0.00547	[50]		−154 ± 43
Rhabdoviruses	1.5704	0.3467	0.2669	0.00384	0.00569	[50]		−111 ± 43
Rhinovirus	1.4950	0.4080	0.2985	0.02308	0.00477	[50]		−197 ± 43
SARS-CoV-2	1.5658	0.3279	0.2901	0.00381	0.00484	[10]		−165 ± 43
Simian virus 5	1.5912	0.3521	0.2563	0.00088	0.00571	[51]		−86 ± 43

**Table 3:** Equations used for finding the thermodynamic properties of viruses. Symbols: *E*—number of electrons transferred to oxygen during complete combustion; *n_J_*—number of atoms of element *J* in the empirical formula of live matter; Δ*_C_H^0^*—standard enthalpy of combustion; Δ*_f_H^0^*(*Bio*)—standard enthalpy of formation of live matter; *S*^0^*_m_*(*Bio*)—standard molar entropy of live matter; *S*^0^*_m_*(*J*)—standard molar entropy of element *J*; *a_J_*—number of atoms per molecule of element *J* in its standard state elemental form (*a_C_* = *a_P_* = *a_S_* = 1 for carbon, phosphorus and sulfur, but *a_H_* = *a_N_* = *a_O_* = 2 for hydrogen, nitrogen and oxygen diatomic molecules [57,58]); Δ*_f_S^0^*(*bio*)—standard entropy of formation of live matter; Δ*_f_G^0^*(*Bio*)—standard Gibbs energy of formation of live matter; CH_1_._7978_O_0_._4831_N_0_._2247_S_0_._0225_—equimolar mixture of amino acids; (Bio)—virions (virus live matter), represented by an empirical formula CH_nH_O_nO_N_nN_P_nP_S_nS_; Δ*_r_G*^0^**—Gibbs energy of growth; *ν*—stoichiometric coefficient. The stoichiometric coefficients for the growth reaction (11) differ between viruses and can be found in the Supplementary Material (Table S1).

Equation Number	Name	Reference
(5)	Combustion electrons	[55,56]
(6)	Patel–Erickson equation	[55,56]
(7)	Hess’ law	[9,10]
(8)	Battley equation	[60]
(9)	Battley formation equation	[60]
(10)	Gibbs equation	[57,58]
(11)	Growth reaction	[9,10]
(12)	Gibbs energy of growth	[57,58]
(13)	Enthalpy uncertainty	[54]
(14)	Entropy uncertainty	[60]

**Table 4:** Outcomes of virus competition, and their Gibbs energies of binding and growth. “Asymmetrical co-infection” is a situation where two viruses are simultaneously present in a multicellular host, but one virus is more numerous. Δ_b_G and Δ_r_G represent Gibbs energies of binding and growth, respectively.

Virus 1	Virus 2	Outcome	Δ_b_G (Virus 1)	Δ_b_G (Virus 2)	Δ_r_G (Virus 1)	Δ_r_G (Virus 2)
Rhinovirus	SARS-CoV-2	Co-infection	−28 to −39	−42.2 to −51.4	−197	−165
Influenza	SARS-CoV-2	Interference	−32.6 to −33.7	−42.2 to −51.4	−83 to −87	−165
Adenovirus	SARS-CoV-2	Asymmetrical co-infection	−45.1 to −45.7	−42.2 to −51.4	−144	−165
Parainfluenza	SARS-CoV-2	Interference	−24.0 to −27.8	−42.2 to −51.4	−86	−165
Influenza	Adenovirus	Interference	−32.6 to −33.7	−45.1 to −45.7	−83 to −87	−165
Influenza	Parainfluenza	Asymmetrical co-infection	−32.6 to −33.7	−24.0 to −27.8	−83 to −87	−86
Parainfluenza 1	Parainfluenza 2	Co-infection	−24.0 to −27.8	−21.7 to −30.4	−86	−86
Influenza	Rhinovirus	Interference	−32.6 to −33.7	−28 to −39	−83 to −87	−197
Rhinovirus	Parainfluenza	Interference	−28 to −39	−24.0 to −27.8	−197	−86

**Table 5:** Gibbs energies and outcomes of pathogen–pathogen–host interactions. “Asymmetrical co-infection” is a situation where two viruses are simultaneously present in a multicellular host, but one virus is more numerous. Δ_b_G_1_ and Δ_b_G_2_ are Gibbs energies of binding of two competing viruses, while Δ_r_G_1_ and Δ_r_G_2_ are their Gibbs energies of growth.

Binding	Growth	Outcome	Examples
Δ_b_G_1_ ≈ Δ_b_G_2_	Δ_r_G_1_ ≈ Δ_r_G_2_	Co-infection	Parainfluenza-1/Parainfluenza-2
Δ_b_G_1_< Δ_b_G_2_	Δ_r_G_1_ ≈ Δ_r_G_2_	Asymmetrical co-infection	Influenza/Parainfluenza
Δ_b_G_1_ ≈ Δ_b_G_2_	Δ_r_G_1_< Δ_r_G_2_	Asymmetrical co-infection	Adenovirus/SARS-CoV-2
			Influenza/Rhinovirus
Δ_b_G_1_< Δ_b_G_2_	Δ_r_G_1_> Δ_r_G_2_	Co-infection	Rhinovirus/SARS-CoV-2
Δ_b_G_1_< Δ_b_G_2_	Δ_r_G_1_< Δ_r_G_2_	Interference	Influenza/SARS-CoV-2
			Parainfluenza/SARS-CoV-2
			Influenza/AdenovirusRhinovirus/Parainfluenza

## Data Availability

Not applicable.

## Supplementary Materials

The following are available online at https://www.mdpi.com/article/10.3390/microorganisms9102060/s1, Text: Growth reactions explanation, Table S1: Stoichiometries of growth of viruses, Table S2: Thermodynamic properties of formation and Growth of viruses, Table S3: Elemental composition of major classes of molecules constituting viruses, Table S4: Elemental composition and thermodynamic properties of the virus growth medium.
