# Supplementary material for: Coinfection and Interference Phenomena Are the Results of Multiple Thermodynamic Competitive Interactions
Source: Microorganisms. 2021 Sep 29;9(10):2060. doi: 10.3390/microorganisms9102060 (PMC8538544; doi:10.3390/microorganisms9102060)
Supplement: Supplementary file 1 [file microorganisms-09-02060-s001.zip › microorganisms-1393573-supplementary.pdf]

Supplementary Material

Coinfection and interference phenomena are the results of multiple thermodynamic competitive interactions

Marko Popovic* ***and*** **Mirjana Minceva**

Biothermodynamics, TUM School of Life Sciences, Technical University of Munich, Maximus-von-Imhof-Forum 2, 85354, Freising, Germany
* Correspondence: author: marko.popovic@tum.de

Growth reactions summarize formation of live matter from a substrate [von Stockar, 2010, 2013a, 2013b]. Table S1 lists the stoichiometric coefficients for growth reactions of the viruses considered in this research. The stoichiometric coefficients are for the growth reaction of the form

$$CH_{1.7978}O_{0.4831}N_{0.2247}S_{0.0225} + O_2 + HPO_4^{2-} + HCO_3^- \rightarrow (Bio) + SO_4^{2-} + H_2O + HCO_3^- + H_2CO_3 \qquad (S.1)$$

where $CH_{1.7978}O_{0.4831}N_{0.2247}S_{0.0225}$ represents amino acids (equation S.1 is the main-text equation 11). (Bio) stands for live matter empirical formula of the form $CH_{nH}O_{nO}N_{nN}P_{nP}S_{nS}$, with coefficients from main-text Table 2. HCO_3^- appears on both sides, because HCO_3^-/H_2CO_3 forms a buffer that regulates the pH. Thus, HCO_3^- is formed or used depending on the number of H^+ ions released by the other reaction participants.

Table S2 gives thermodynamic properties of formation and growth of the viruses considered in this research. Standard molar enthalpy, $\Delta_r H^0$, and entropy, $\Delta_r S^0$, of growth were calculated using the equations

$$\Delta_r H^0 = \sum_{products} \nu\, \Delta_f H^0 - \sum_{reactants} \nu\, \Delta_f H^0 \qquad (S.2)$$

$$\Delta_r S^0 = \sum_{products} \nu\, S_m^o - \sum_{reactants} \nu\, S_m^o \qquad (S.3)$$

where $\Delta_f H^0$ is standard enthalpy of formation, S^0_m standard molar entropy and ν stoichiometric coefficient from Table S1 [Atkins and de Paula, 2014, 2011]. The summations are over all reactants and products of the growth reaction (S.1). The uncertainties in $\Delta_r H^0$ and $\Delta_r S^0$ were found by error propagation, starting from uncertainties in $\Delta_f H^0$ and S^0_m.

Table S.3 lists elemental compositions and molar masses of the major molecular constituents of viruses: RNA, DNA, proteins, carbohydrates and lipids. The values were taken from [Popovic and Minceva, 2020a]. This data was combined with molecular composition of viruses, using main-text equation 3, to find the elemental compositions of viruses.

Table S.4 gives chemical formulas and standard thermodynamic properties of the growth medium components. These properties were combined with thermodynamic properties of viruses [$\Delta_f H^0(Bio)$, $S^0_m(Bio)$ and $\Delta_f G^0(Bio)$ from Table S2] and stoichiometry of growth (Table S1) to find thermodynamic properties of virus growth ($\Delta_r H^0$, $\Delta_r S^0$ and $\Delta_r G^0$), using equations S.2, S.3 and main-text equation 12. The results are given in Table S2 and main-text Table 3.

References

Atkins, P. and de Paula, J. (2014). *Physical Chemistry: Thermodynamics, Structure, and Change, 10th ed.*, New York: W.H. Freeman and Company.

Atkins, P. and de Paula, J. (2011). *Physical Chemistry for the Life Sciences, 2nd ed.*, New York: W.H. Freeman and Company.

Popovic, M., & Minceva, M. (2020a). A thermodynamic insight into viral infections: do viruses in a lytic cycle hijack cell metabolism due to their low Gibbs energy?. *Heliyon*, *6*(5), e03933. https://doi.org/10.1016/j.heliyon.2020.e03933

Vieillard, P. and Tardy, Y. (1984). Thermochemical properties of phosphates. In: *Phosphate Minerals*, Nriagu, J.O., Moore, P.B., eds., Berlin: Springer-Verlag, pp. 171-198.

Von Stockar, U. (2010). Biothermodynamics of live cells: a tool for biotechnology and biochemical engineering. *Journal of Non-Equilibrium Thermodynamics*, 35(4), 415-475. https://doi.org/10.1515/jnetdy.2010.024.

Von Stockar, U. (2013a). Live cells as open non-equilibrium systems. In: *Biothermodynamics: The Role of Thermodynamics in Biochemical Engineering,* Urs von Stockar, ed., Lausanne: EPFL Press, pp. 399-421. https://doi.org/10.1201/b15428-23

Von Stockar, U. (2013b). Biothermodynamics of live cells: Energy disipation and heat generation in cellular cultures. In *Biothermodynamics: The Role of Thermodynamics in Biochemical Engineering,* Urs von Stockar, ed., Lausanne: EPFL Press, pp. 475-534. https://doi.org/10.1201/b15428-26

Table S1. Stoichiometries of growth of viruses. The stoichiometric coefficients are for the growth reaction $CH_{1.7978}O_{0.4831}N_{0.2247}S_{0.0225}$ + O_2 + HPO_4^{2-} + HCO_3^- → (Bio) + SO_4^{2-} + H_2O + HCO_3^- + H_2CO_3, where $CH_{1.7978}O_{0.4831}N_{0.2247}S_{0.0225}$ represents amino acids. (Bio) stands for live matter empirical formula of the form $CH_{nH}O_{nO}N_{nN}P_{nP}S_{nS}$, with coefficients from main-text Table 2. The viruses marked with an asterisk are enveloped viruses. For enveloped viruses, lipids were not included into calculation, since they are taken from the host cell during budding and not synthesized at the ribosomes [Popovic and Minceva, 2020b].

Name	Composition reference	Reactants					Products				
		Amino acid	O_2	HPO_4^{2-}	HCO_3^-	→	Bio	SO_4^{2-}	H_2O	HCO_3^-	H_2CO_3
Adenovirus	[Riedel et al., 2019]	1.25226	0.31707	0.00997	0.02531	→	1.00000	0.02263	0.09639	0.00000	0.27757
Adenovirus	[Knight, 1975]	1.25226	0.31707	0.00997	0.02531	→	1.00000	0.02263	0.09639	0.00000	0.27757
Coliphages T2, T4, T6	[Knight, 1975]	1.38163	0.52576	0.04868	0.00000	→	1.00000	0.02812	0.18563	0.04112	0.34051
Enterovirus	[Riedel et al., 2019]	1.32815	0.44323	0.02308	0.00401	→	1.00000	0.02508	0.12772	0.00000	0.33216
Equine encephalomyelitis*	[Knight, 1975]	1.23801	0.30097	0.00619	0.03177	→	1.00000	0.02207	0.08735	0.00000	0.26979
Fowl plague*	[Knight, 1975]	1.15326	0.20071	0.00189	0.03668	→	1.00000	0.02023	0.07336	0.00000	0.18995
Herpes simplex*	[Knight, 1975]	1.22296	0.28459	0.00887	0.02631	→	1.00000	0.02203	0.09231	0.00000	0.24927
Influenza*	[Riedel et al., 2019]	1.13477	0.17813	0.00071	0.03820	→	1.00000	0.01981	0.06980	0.00000	0.17297
Influenza*	[Knight, 1975]	1.14355	0.18724	0.00071	0.03850	→	1.00000	0.01996	0.07034	0.00000	0.18205
Orthomyxoviruses*	[Riedel et al., 2019]	1.14036	0.18443	0.00088	0.03808	→	1.00000	0.01992	0.07050	0.00000	0.17844
Paramyxoviruses*	[Riedel et al., 2019]	1.14036	0.18443	0.00088	0.03808	→	1.00000	0.01992	0.07050	0.00000	0.17844
Picornaviruses	[Riedel et al., 2019]	1.32815	0.44323	0.02308	0.00401	→	1.00000	0.02508	0.12772	0.00000	0.33216
Poliovirus	[Riedel et al., 2019]	1.32815	0.44323	0.02308	0.00401	→	1.00000	0.02508	0.12772	0.00000	0.33216
Poxviruses*	[Riedel et al., 2019]	1.21641	0.26534	0.00241	0.03793	→	1.00000	0.02137	0.07832	0.00000	0.25434
Reoviruses, Rotaviruses, and Caliciviruses	[Riedel et al., 2019]	1.26324	0.34079	0.01091	0.02400	→	1.00000	0.02291	0.09865	0.00000	0.28724
Rhabdoviruses*	[Riedel et al., 2019]	1.18779	0.24214	0.00384	0.03432	→	1.00000	0.02100	0.07947	0.00000	0.22211
Rhinovirus	[Riedel et al., 2019]	1.32815	0.44323	0.02308	0.00401	→	1.00000	0.02508	0.12772	0.00000	0.33216
SARS-CoV-2*	[Popovic and Minceva, 2020b]	1.29093	0.36323	0.00381	0.04072	→	1.00000	0.02417	0.06809	0.00000	0.33165
Simian virus 5*	[Knight, 1975]	1.14036	0.18443	0.00088	0.03808	→	1.00000	0.01992	0.07050	0.00000	0.17844

Table S2: Thermodynamic properties of formation and Growth of viruses. Symbols: $\Delta_f H^0(Bio)$ - standard enthalpy of formation of live matter; $S^0{}_m(Bio)$ - standard molar entropy of live matter; $\Delta_f G^0(Bio)$ – standard Gibbs energy of formation of live matter; $\Delta_r H^0$ - enthalpy of growth; $\Delta_r S^0$ - entropy of growth; $\Delta_r G^0$ - Gibbs energy of growth

Name	Composition reference	$\Delta_f H^0$(bio) (kJ/C-mol)	$S^0{}_m$(bio) (J/C-mol K)	$\Delta_f G^0$(bio) (kJ/C-mol)	$\Delta_r H^0$ (kJ/C-mol)	$\Delta_r S^0$ (J/C-mol K)	$\Delta_r G^0$ (kJ/C-mol)
Adenovirus	[Riedel et al., 2019]	-73 ± 29	31.4 ± 6.2	-32 ± 30	-150 ± 42	-22.4 ± 9.5	-144 ± 43
Adenovirus	[Knight, 1975]	-73 ± 29	31.4 ± 6.2	-32 ± 30	-150 ± 42	-22.4 ± 9.5	-144 ± 43
Coliphages T2, T4, T6	[Knight, 1975]	-108 ± 28	33.6 ± 6.6	-65 ± 28	-242 ± 41	-43.9 ± 9.8	-230 ± 43
Enterovirus	[Riedel et al., 2019]	-90 ± 29	32.7 ± 6.4	-48 ± 29	-207 ± 41	-35.5 ± 9.6	-197 ± 43
Equine encephalomyelitis*	[Knight, 1975]	-72 ± 29	31.5 ± 6.2	-32 ± 30	-143 ± 42	-21.2 ± 9.5	-137 ± 43
Fowl plague*	[Knight, 1975]	-76 ± 29	31.8 ± 6.3	-35 ± 29	-97 ± 42	-13.6 ± 9.5	-93 ± 43
Herpes simplex*	[Knight, 1975]	-75 ± 29	31.6 ± 6.2	-34 ± 29	-135 ± 42	-20.1 ± 9.5	-129 ± 43
Influenza*	[Riedel et al., 2019]	-77 ± 29	31.9 ± 6.3	-36 ± 29	-87 ± 42	-11.8 ± 9.5	-83 ± 43
Influenza*	[Knight, 1975]	-76 ± 29	31.8 ± 6.3	-34 ± 29	-91 ± 42	-12.3 ± 9.5	-87 ± 43
Orthomyxoviruses*	[Riedel et al., 2019]	-76 ± 29	31.9 ± 6.3	-35 ± 29	-90 ± 42	-12.2 ± 9.5	-86 ± 43
Paramyxoviruses*	[Riedel et al., 2019]	-76 ± 29	31.9 ± 6.3	-35 ± 29	-90 ± 42	-12.2 ± 9.5	-86 ± 43
Picornaviruses	[Riedel et al., 2019]	-90 ± 29	32.7 ± 6.4	-48 ± 29	-207 ± 41	-35.5 ± 9.6	-197 ± 43
Poliovirus	[Riedel et al., 2019]	-90 ± 29	32.7 ± 6.4	-48 ± 29	-207 ± 41	-35.5 ± 9.6	-197 ± 43
Poxviruses*	[Riedel et al., 2019]	-67 ± 30	31.1 ± 6.1	-27 ± 30	-127 ± 42	-17.5 ± 9.4	-122 ± 43
Reoviruses, Rotaviruses, and Caliciviruses	[Riedel et al., 2019]	-77 ± 29	31.8 ± 6.3	-36 ± 29	-161 ± 42	-25.2 ± 9.5	-154 ± 43
Rhabdoviruses*	[Riedel et al., 2019]	-75 ± 29	31.7 ± 6.2	-34 ± 29	-116 ± 42	-16.8 ± 9.5	-111 ± 43
Rhinovirus	[Riedel et al., 2019]	-90 ± 29	32.7 ± 6.4	-48 ± 29	-207 ± 41	-35.5 ± 9.6	-197 ± 43
SARS-CoV-2*	[Popovic and Minceva, 2020b]	-71 ± 30	31.7 ± 6.2	-30 ± 30	-173 ± 42	-26.2 ± 9.5	-165 ± 43
Simian virus 5*	[Knight, 1975]	-76 ± 29	31.9 ± 6.3	-35 ± 29	-90 ± 42	-12.2 ± 9.5	-86 ± 43

Table S3: Elemental composition of major classes of molecules constituting viruses. Elemental composition is given in the form of empirical formulas: $CH_{nH}O_{nO}N_{nN}P_{nP}S_{nS}$. Since the empirical formulas are per C-mole, $n_C = 1$. Data taken from [Popovic and Minceva, 2020a].

Class	n_H	n_O	n_N	n_P	n_S	Mr (g/C-mol)
RNA	1.23160	0.76101	0.39670	0.10500	0.00000	34.216
DNA	1.25548	0.58400	0.37961	0.10219	0.00000	31.082
Protein	1.56922	0.30849	0.27079	0.00000	0.00611	22.492
Carbohydrate	2.00000	1.00000	0.00000	0.00000	0.00000	30.000
Lipid	1.92157	0.11765	0.00000	0.00000	0.00000	15.804

Table S4: Elemental composition and thermodynamic properties of the virus growth medium. Standard thermodynamic properties of the mixture of amino acids were estimated from its elemental composition using the Battley equation. $\Delta_f H°$ - standard enthalpy of formation; $S°_m$ standard molar entropy; $\Delta_f G°$ - standard Gibbs energy of formation.

Name	Formula	$\Delta_f H°$ (kJ/mol)	$S°_m$ (J/mol K)	$\Delta_f G°$ (kJ/mol)	Reference
Amino acid	$CH_{1.7978}O_{0.4831}N_{0.2247}S_{0.0225}$	-107±30	36±7	-60±30	Battley equation
Oxygen	O_2 (g)	0	205.138	0	[Atkins and de Paula, 2014]
Hydrogenphosphate	HPO_4^{2-} (aq)	-1292.14	8.4	-1089.26	[Vieillard and Tardy, 1984]
Bicarbonate	HCO_3^- (aq)	-691.99	91.2	-586.77	[Atkins and de Paula, 2014]
Sulfate	SO_4^{2-} (aq)	-909.27	20.1	-744.53	[Atkins and de Paula, 2014]
Water	H_2O (l)	-285.83	69.91	-237.13	[Atkins and de Paula, 2014]
Carbonic acid	H_2CO_3 (aq)	-699.65	187.4	-623.08	[Atkins and de Paula, 2014]
